# Progress on intelligent metasurfaces for signal relay, transmitter, and processor

**DOI:** 10.1038/s41377-024-01729-2

**Published:** 2025-02-25

**Authors:** Chao Qian, Longwei Tian, Hongsheng Chen

**Affiliations:** 1 ZJU-UIUC Institute, Interdisciplinary Center for Quantum Information, State Key Laboratory of Extreme Photonics and Instrumentation, Zhejiang University, Hangzhou, China; 2 ZJU-Hangzhou Global Science and Technology Innovation Center, Key Lab. of Advanced Micro/Nano Electronic Devices & Smart Systems of Zhejiang, Zhejiang University, Hangzhou, China; 3 Jinhua Institute of Zhejiang University, Zhejiang University, Jinhua, China; 4 Shanghai Key Laboratory of Navigation and Location-Based Services, Shanghai Jiao Tong University, Shanghai, China

**Keywords:** Metamaterials, Other photonics

## Abstract

Pursuing higher data rate with limited spectral resources is a longstanding topic that has triggered the fast growth of modern wireless communication techniques. However, the massive deployment of active nodes to compensate for propagation loss necessitates high hardware expenditure, energy consumption, and maintenance cost, as well as complicated network interference issues. Intelligent metasurfaces, composed of a number of subwavelength passive or active meta-atoms, have recently found to be a new paradigm to actively reshape wireless communication environment in a green way, distinct from conventional works that passively adapt to the surrounding. In this review, we offer a unified perspective on how intelligent metasurfaces can facilitate wireless communication in three manners: signal relay, signal transmitter, and signal processor. We start by the basic modeling of wireless channel and the evolution of metasurfaces from passive, active to intelligent metasurfaces. Integrated with various deep learning algorithms, intelligent metasurfaces adapt to cater for the ever-changing environments without human intervention. Then, we overview specific experimental advancements using intelligent metasurfaces. We conclude by identifying key issues in the practical implementations of intelligent metasurfaces, and surveying new directions, such as gain metasurfaces and knowledge migration.

## Abstract (web summary)

This article offers a unified perspective on how intelligent metasurfaces facilitate wireless communication in the ways of signal relay, transmitter, and processor.

## Graphical abstract

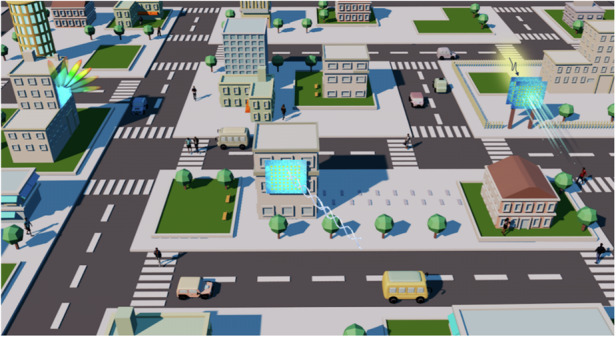

## Introduction

Since the first radio transmission sent across the Atlantic Ocean in 1901, wireless communication has started to attract widespread attention from academia to industry. After a century, the world of wireless networks has undergone a remarkable transformation, from early brick-sized cell phones to present-day seamless streaming. Pursuing higher data rate with limited spectral resources is an eternal goal, especially within the context of the growing global usage of smart devices and augmented reality applications. The fifth generation (5G) is a new technology to dramatically increase the network capacity by integrating massive multiple-input multiple-output (MIMO), millimeter wave (mmWave) communication, and orthogonal frequency-division multiplexing^1^. To this end, conventional wisdom requires the dense deployment of a large number of active nodes, such as base stations and antenna arrays, to shorten the communication distance. However, this inevitably brings increased hardware expenditure, energy consumption, maintenance cost, and complicated network interference issues. In addition, migrating the communication band from sub-6 GHz to mmWave will necessitate more complex active nodes to offset higher propagation loss. Therefore, developing another spectral and energy efficient but low-cost framework for the forthcoming 5G and future beyond 5G/the sixth-generation (6G) wireless networks becomes imperative^2^.

Intelligent metasurfaces have recently emerged as a promising paradigm with huge potential to eliminate the extensive use of transmit radio-frequency (RF) chains and reduce the hardware cost^3–7^. The basic concept of intelligent metasurface stems from electromagnetism and optics decades ago, which are artificial media comprising subwavelength unit cells/meta-atoms^8,9^. By carefully designing the geometries and spatial distribution, metasurfaces can be endowed with unprecedented abilities to manipulate the polarization, amplitude, and phase of electromagnetic (EM) waves at interface. Compared with conventional three-dimensional (3D) metamaterials, the features of compactness, lightweight, better integration, and lower loss make metasurfaces rapidly gain commercial popularity and the operation regime has been generalized to other physical systems^10–16^. Looking back, two-decade development of metasurfaces has evolved from passive, reconfigurable/tunable/active, to intelligent modalities^17–19^. To be more exactly, intelligent metasurfaces shall be integrated with intelligent algorithms to automatically satisfy user demand and cater for ever-changing environment^20–25^. This is different from passive and reconfigurable metasurfaces because they have the ability of engineering EM waves but lacking of intelligence. In the community of wireless communication, intelligent metasurfaces, also known as the terminologies of intelligent reflecting surfaces (IRS) and reconfigurable intelligent surfaces (RIS), has started to attract extensive attention about five years ago. In their understanding, both passive and active metasurfaces are generally regarded as IRS and RIS. This is the conceptual difference across different communities. In this Review, we will use the term of intelligent metasurfaces and interpret specific concept inside to strengthen the understanding.

Generally speaking, intelligent metasurfaces provide a green and low-cost route to reshape wireless communication channel as desired, while conventional works have to passively adapt to the uncontrollable environments^26–28^. For example, in a dead zone, intelligent metasurfaces can be used as a signal relay to create a virtual line-of-sight link. For on-demand signal propagation, intelligent metasurfaces can automatically control all meta-atoms to strengthen the desired signal to improve the channel rank condition. To be specific, intelligent metasurfaces feature various practical advantages for implementation. The reflective meta-atoms can globally manipulate the incident signals in a customized way without complex RF links, thus can be executed with much lower hardware/energy cost compared to traditional antenna arrays. Furthermore, intelligent metasurfaces can be readily mounted on or removed from environmental settings and non-invasively integrated to the wireless networks, e.g., decorated on the walls of buildings and packaged by aerial platforms. Far beyond wave manipulation, intelligent metasurfaces have the potential to on-site process and compute signals at the physical layer, such as dimensionality reduction and feature extraction^29–34^. These advantages enable intelligent metasurfaces become a hot topic, but also leaving many open practical challenges.

**Fig. 1:**
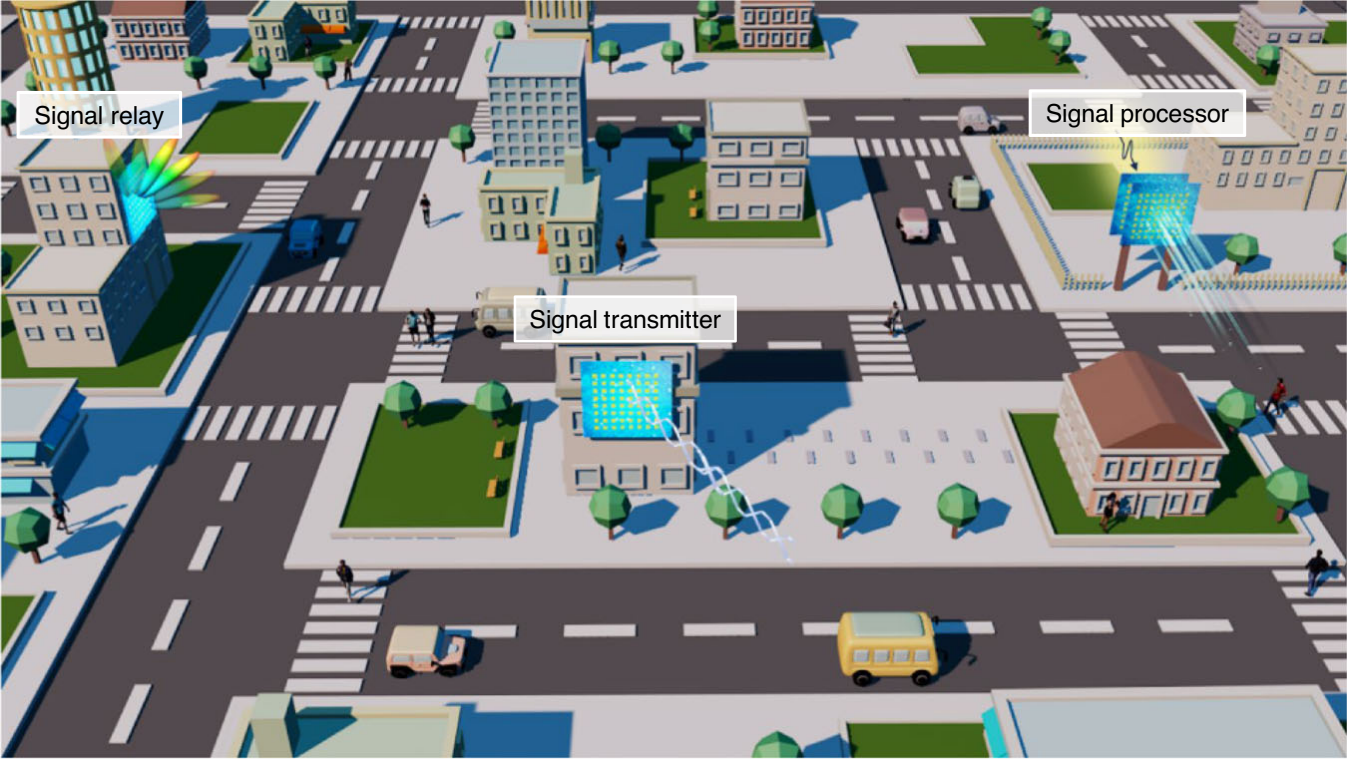
Application scenarios of intelligent metasurface in wireless communication Intelligent metasurfaces can be flexibly distributed among city infrastructures, which can be used for three typical purposes: signal relay, signal transmitter, and signal processor

In this Review, we provide an overview on the recent advancements of intelligent metasurfaces for the free management of entire wireless communication environment (Fig. 1). We start from channel modeling and survey active approaches to achieve tunable metasurfaces at microwave. We pay special attention to deep learning algorithms for forward prediction of EM scattering and inverse design of metasurface distribution, which is a key to reach true intelligence. Throughout, we provide the experimental implementations on how to reshape wireless communication environment and, more interestingly, underscore how to physically process signal. In the last part, standing from the perspective of electromagnetics, we point out the possible future directions where substantial impact is expected in the coming years and imminent challenges that hinder the further off-the-shelf applications.

## Intelligent metasurfaces-assisted wireless channel model

### A. Cascaded channel model

**Fig. 2:**
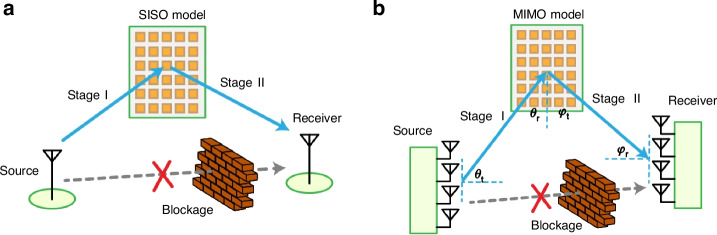
Intelligent metasurface assisted wireless channel model. **a** Cascaded channel model for a general intelligent metasurfaces assisted single-input single-output (SISO) wireless communication system. **b** Sparse angular channel model for intelligent metasurfaces assisted Multiple-input multiple-output (MIMO) wireless communication system

Figure 2a shows a general intelligent metasurfaces-assisted single-input single-output (SISO) wireless communication system^35–37^. We focus on the wireless channel reflected from the signal source to the receiver through an intelligent metasurfaces and divide it into two stages: Stage I, from signal source to the metasurfaces, and Stage II, from the metasurfaces to receiver.

In stage I, let $x\left(t\right)\in {\mathbb{C}}$ denote the equivalent baseband transmit signal in narrow band, the intelligent metasurfaces contain an array of reflection elements, each provides a possible channel link. The signal impinging on metasurface element *n* is

1 $${y}_{i,n}\left(t\right)={\alpha }_{n}^{\left(1\right)}{e}^{-j{\varphi }_{n}^{\left(1\right)}}x\left(t\right)$$

where ${\alpha }_{n}^{\left(1\right)}$ denotes the amplitude attenuation and ${\varphi }_{n}^{\left(1\right)}$ denotes the phase delay through channel propagation in stage I.

Then, each element reflects the signal with additional attenuation ${\beta }_{n}$ and phase delay ${\theta }_{n}$ as

2 $${y}_{o,n}\left(t\right)={\beta }_{n}{e\,}^{j{\theta }_{n}}{y}_{i,n}\left(t\right)={\beta }_{n}{\alpha }_{n}^{\left(1\right)}{e\,}^{j\left(-{\varphi }_{n}^{\left(1\right)}+{\theta }_{n}\right)}x\left(t\right)$$

In stage II, the reflected signal from IRS to the receiver goes through a similar channel propagation as

3 $${y}_{r,n}\left(t\right)={\alpha }_{n}^{\left(2\right)}{e\,}^{j{\varphi }_{n}^{\left(2\right)}}{y}_{o,n}\left(t\right)={\beta }_{n}{\alpha }_{n}^{\left(1\right)}{\alpha }_{n}^{\left(2\right)}{e\,}^{j\left(-{\varphi }_{n}^{\left(1\right)}+{\varphi }_{n}^{\left(2\right)}+{\theta }_{n}\right)}x\left(t\right)$$

where ${\alpha }_{n}^{\left(2\right)}$ denotes the amplitude attenuation and ${\varphi }_{n}^{\left(2\right)}$ denote phase delay through channel propagation in stage II.

Denote ${h}_{n}={\alpha }_{n}^{\left(1\right)}{e}^{-j{\varphi }_{n}^{\left(1\right)}}$ and ${g}_{n}={\alpha }_{n}^{\left(2\right)}{e}^{-j{\varphi }_{n}^{\left(2\right)}}$, the corresponding received signal is

4 $${y}_{r,n}\left(t\right)={g}_{n}^{* }{\beta }_{n}{e\,}^{j{\theta }_{n}}{h}_{n}\cdot x\left(t\right)$$

Hence the total received signal at receiver is

5 $${y}_{r}\left(t\right)=\left(\mathop{\sum }\limits_{n=1}^{N}{g}_{n}^{* }{\beta }_{n}{e\,}^{j{\theta }_{n}}{h}_{n}\right)x\left(t\right)={{\boldsymbol{g}}}^{H}{\boldsymbol{\Gamma }}{\boldsymbol{h}}\cdot x\left(t\right)$$

where the equivalent channel coefficients are represented in vector and matrix form as ${\boldsymbol{h}}={\left[{h}_{1},{h}_{2}\cdots {h}_{N}\right]}^{T}$, ${\boldsymbol{g}}={\left[{g}_{1},{g}_{2}\cdots {g}_{N}\right]}^{T}$ and ${\boldsymbol{\Gamma }}={diag}\left({\beta }_{1}{e}^{j{\theta }_{1}},{\beta }_{2}{e}^{j{\theta }_{2}}\cdots {\beta }_{N}{e}^{j{\theta }_{N}}\right)$. Hence the cascaded SISO channel model is ${{\boldsymbol{g}}}^{H}{\boldsymbol{\Gamma }}{\boldsymbol{h}}$.

## B. Sparse angular channel model

In the scenario of intelligent metasurfaces-assisted mmWave and terahertz systems, signal attenuation along the path increases significantly as the communication frequency increases, so large-scale MIMO systems have been widely applied to utilize space diversity gain versus path loss^38^. In this case, the cascade channel model can be transformed to an angular domain sparse channel model^39–41^, as shown in Fig. 2b. Similar to SISO, the channel reflected by the metasurfaces can be divided into two stages, namely from signal source to the metasurfaces, and from the metasurfaces to receiver.

Assume that the number of antennas at the transmitter, receiver, and metasurface elements are ${N}_{T}$, ${N}_{R}$ and ${N}_{F}$, respectively. The number of path clusters between the transmitter and metasurfaces is ${P}_{T}$, and the number of path clusters between the receiver and metasurfaces is ${P}_{R}$. In stage I, let $x\left(t\right)\in {\mathbb{C}}$ denote the equivalent baseband transmit signal in narrow band, with the definition of directional vector ${{\boldsymbol{a}}}_{N}\left(\theta \right)=\frac{1}{\sqrt{N}}{\left[1,{e}^{j\frac{2\pi d}{\lambda }\sin \theta }\cdots ,{e}^{j\frac{2\pi d}{\lambda }\left(N-1\right)\sin \theta }\right]}^{T}$, the signal from the transmit antenna array impinging on IRS elements is

6 $${{\boldsymbol{y}}}_{i}\left(t\right)=\sqrt{\frac{{N}_{T}{N}_{F}}{{P}_{T}}}\mathop{\sum }\limits_{p=1}^{{P}_{T}}{\alpha }_{p}^{\left(1\right)}{{\boldsymbol{a}}}_{{N}_{F}}\left({\theta }_{r}^{\left(p\right)}\right){{\boldsymbol{a}}}_{{N}_{T}}^{H}\left({\theta }_{t}^{\left(p\right)}\right)\cdot x\left(t\right)$$

where ${\alpha }_{p}^{\left(1\right)}$ denotes the amplitude attenuation, ${\theta }_{r}^{\left(p\right)}$ and ${\theta }_{t}^{\left(p\right)}$ denote the angle of arrival (AoA) and the angle of departure (AoD) from the source to metasurfaces in the *p*th path in stage I, respectively. The coefficient $\sqrt{\frac{{N}_{T}{N}_{F}}{{P}_{T}}}$ is for amplitude normalization.

Then, each element reflects the signal with additional attenuation ${\beta }_{n}$ and phase delay ${\theta }_{n}$ as

7 $${{\boldsymbol{y}}}_{o}\left(t\right)={\boldsymbol{\Gamma }}{{\boldsymbol{y}}}_{i}\left(t\right)={\boldsymbol{\Gamma }}\left[\sqrt{\frac{{N}_{T}{N}_{F}}{{P}_{T}}}\mathop{\sum }\limits_{p=1}^{{P}_{T}}{\alpha }_{p}^{\left(1\right)}{{\boldsymbol{a}}}_{{N}_{F}}\left({\theta }_{r}^{\left(p\right)}\right){{\boldsymbol{a}}}_{{N}_{T}}^{H}\left({\theta }_{t}^{\left(p\right)}\right)\right]\cdot x\left(t\right)$$

where ${\boldsymbol{\Gamma }}={diag}\left({\beta }_{1}{e}^{j{\theta }_{1}},{\beta }_{2}{e}^{j{\theta }_{2}}\cdots {\beta }_{N}{e}^{j{\theta }_{N}}\right)$ is the reflection coefficients by the metasurfaces.

In stage II, the reflected signal from IRS to the receiver goes through a similar channel propagation as

8 $${{\boldsymbol{y}}}_{r}\left(t\right)={\left[\sqrt{\frac{{N}_{F}{N}_{R}}{{P}_{R}}}\mathop{\sum }\limits_{q=1}^{{P}_{R}}{\alpha }_{q}^{\left(2\right)}{{\boldsymbol{a}}}_{{N}_{R}}\left({\varphi }_{r}^{\left(q\right)}\right){{\boldsymbol{a}}}_{{N}_{F}}^{H}\left({\varphi }_{t}^{\left(q\right)}\right)\right]}^{H}{{\boldsymbol{y}}}_{o}\left(t\right)$$

where ${\alpha }_{q}^{\left(2\right)}$ denotes the amplitude attenuation, ${\varphi }_{r}^{\left(q\right)}$ and ${\varphi }_{t}^{\left(q\right)}$ denote AoA and AoD from the metasurfaces to the receiver in the *q*th path in stage II, respectively. The coefficient $\sqrt{\frac{{N}_{F}{N}_{R}}{{P}_{R}}}$ is for amplitude normalization.

Denote ${\boldsymbol{H}}={\sum }_{p=1}^{{P}_{T}}{\alpha }_{p}^{\left(1\right)}{{\boldsymbol{a}}}_{{N}_{F}}\left({\theta }_{r}^{\left(p\right)}\right){{\boldsymbol{a}}}_{{N}_{T}}^{H}\left({\theta }_{t}^{\left(p\right)}\right)$ and ${\boldsymbol{G}}={\sum }_{q=1}^{{P}_{R}}{\alpha }_{q}^{\left(2\right)}{{\boldsymbol{a}}}_{{N}_{R}}\left({\varphi }_{r}^{\left(q\right)}\right){{\boldsymbol{a}}}_{{N}_{F}}^{H}\left({\varphi }_{t}^{\left(q\right)}\right)$, the corresponding signal at receiver is

9 $${{\boldsymbol{y}}}_{r}\left(t\right)={{\boldsymbol{G}}}^{H}{\boldsymbol{\Gamma }}{\boldsymbol{H}}\cdot x\left(t\right)$$

Hence the sparse angular channel model can be modeled as ${{\boldsymbol{G}}}^{H}{\boldsymbol{\Gamma }}{\boldsymbol{H}}$.

## Evolution of metasurfaces: from passive, active, to intelligent metasurfaces

At the end of the last century, the attempts to artificially construct unique materials that do not exist in nature have greatly triggered the advent of metamaterials^42^. Canonical examples include negative-index media, hyperbolic materials, zero-index media and, more broadly, materials with bespoke permittivity and permeability^43^. Metamaterials are composed of subwavelength meta-atoms in 3D lattice that resonantly interact with the electric and magnetic fields of the incident EM waves. They greatly reshape the landscape of natural materials and defy well-documented physical laws. However, the further development of metamaterials is hindered by the strong dispersion, bulky volume, and high insertion loss. In this context, metasurfaces, as the planar equivalence of metamaterials, have found to be a promising candidate to control the polarization, phase, and amplitude of EM waves. The initial purpose of metasurfaces have generalized the Snell’s laws with the introduction of gradient phase and then quickly led to a panoply of novel applications, such as anomalous reflection, polarization converter, and dispersion engineering.

**Fig. 3:**
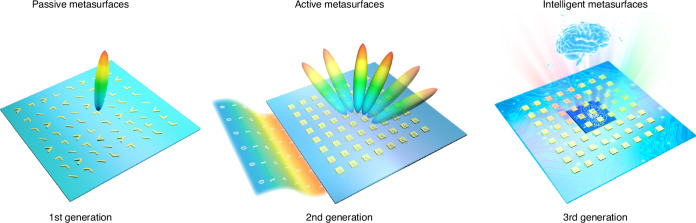
Three generations of metasurfaces: passive, active, to intelligent metasurfaces. The distinct features of intelligent metasurfaces lie in the ability of quickly satisfying user demand and catering for changing environments without human intervention

Historically, metasurfaces have gone through three main generations: passive, active and intelligent metasurfaces (Fig. 3). Initial metasurfaces were studied to provide an individual EM response for a single incident wave with specific frequency, incident angle, and polarization. The basic meta-atom can be made of diverse material compositions with different geometries and distributions, e.g., dielectric and semiconducting, allowing for the desired response to EM waves. In microwave, metasurfaces, similar to frequency selective surfaces, are typically made of metallic patches and dielectric substrate in multi-layer configurations with the features of lightweight, easy fabrication, and the ability to control wave propagation along the surface and in free space. Through flexible design, they have been extensively studied in filters, selectors, absorbers, antenna radomes, and more^19^. However, once fabricated, the functionalities are set in stone, making it difficult for dynamic applications.

New insights into metasurfaces with tunable functionalities have shifted research interests from the discovery of exciting optical phenomena towards practical applications^44^. Tunable, reconfigurable, programmable, and active metasurfaces are capable of dynamically manipulating EM waves, getting rid of the constraints of passive and static systems. On this basis, coding, and information metasurfaces are then proposed to bridge the digital information world and physical world. The tuning strategies can be generally classified into two major categories. One is mechanical reconfiguration-based structural variations, such as modifying the lattice constants, meta-atom shapes, and spatial arrangements under various external stimuli. Such strategy is applicable across different frequencies but often limited to a slow response speed^45,46^. The other is in conjunction with active components, including varactor diodes, liquid crystals, phase change materials, and two-dimensional (2D) materials, where the sensitivity of optical properties to external stimuli enables the dynamic control^47,48^. In contrast to mechanical actuation, this tuning strategy can operate at a faster speed but is often within narrow band due to material dispersion. Although reconfigurable metamaterials have been studied and improve the performance of established technologies, they often work with external assistance and trial-and-error manner to meet user-specific requirements, strongly hindering real-time applications.

The third generation, intelligent metasurfaces, has recently garnered extensive attention due to the distinct features of decision-making, self-programming, and executing a series of successive tasks automatically^20,31^. Nowadays, intelligent metasurfaces are a fashionable area of research, making it difficult to separate the hype from the true utility. Compared with the second generations—reconfigurable metasurfaces, intelligent metasurfaces build upon but different from them. To put it simply, reconfigurable metasurfaces provide hardware basis for intelligent metasurfaces, and intelligent algorithms, especially deep learning, provide software basis. Conventional reconfigurable metasurfaces endow meta-devices with reconfigurability, but they are encountered with a knotty issue of how to quickly and automatically cater for customer-defined functionalities and on-site requirements. For a given EM goal, reconfigurable metasurfaces still need time-consuming bottom-up searches and repeated manual control, which limits the application scope. The fast development of deep learning offers a data-driven method to inversely bridge the scattering features (intensity, near-field, far-field, etc.) from metasurface geometries and distributions. In practice, intelligent metasurfaces are able to automatically and quickly satisfy user demand and cater for changing environments. For example, metasurfaces based self-adaptive invisibility cloak has been recently demonstrated. Intelligent metasurfaces-based cloak incorporates with three modules, perception, decision, and execution, mimicking the three key camouflage components of chameleon, i.e., photon-sensitive cells, a central nervous system, and pigment cells. The reflection property of each unit cell can be independently changed by applying different bias voltages to the varactor diode. Driven by a pretrained artificial neural network, the metasurface cloak can respond rapidly, on a millisecond timescale, to the ever-changing incident wave and surrounding background, as demonstrated by an intelligent cloaked vehicle^20^. Its intellectualization will lead to other interesting applications, such as adaptive focusing, wireless communications, wave-based computing, and many more^49–51^.

## Deep learning agents

To make metasurfaces automatically cater for wireless communication demands, deep learning is an important algorithm to be integrated as a high-speed computing core to assist, accelerate and ultimately automate metasurface design, system optimization, and automation control^52–54^. For this inverse design, its solution cannot be directly evaluated, instead requiring the exploration of a suitable solution from a formidably large design space. Conventionally, people can iteratively search for an acceptable metasurface via a gargantuan number of forward simulations, in conjunction with heuristic algorithms and empirical guidance^55–57^. However, such simulations are time-consuming, limiting the cases to simple and small structures. As a data-driven method, deep learning can be trained to predict, classify, and cluster data with strong nonlinear fitting ability^58^. It has been found to capture complex, nuanced relationships between EM scattering, environment and metasurfaces without a model-based theory. In the past decade, its awakened wave has swept from the mainstream applications of image recognition and language translation to the disciplines of electromagnetism and wireless communications. The main advantage of deep learning lies in its agnosticism about the nature of training data, allowing it to capture the relationship between conceptually distant causes and effects^59–62^.

Deep learning algorithms are many, from basic multilayer perceptron to large language model. In general, they can be divided into discriminative and generative networks. Most of them are inspired from computer science, and many review papers have introduced the basic principle and applications of them. For example, recurrent neural network feeds the network outputs back into the input layer, having a memory that accounts for the past state of the system, which makes them useful to model time-sequential systems. So far, we have seen extensive literature across diversified metasurfaces, antennas, and photonic crystals^52–54^. In this Review, we pay attention to more novel algorithms that exclusively match well with the background of metasurfaces and wireless communications.

### Non-uniqueness issue

In wireless communications, we aim to construct a desired EM environment by inversely designing metasurface deployment, distribution and, more generally, all physical variables that affect the EM environment. Such inverse design is much challenging due to the non-convex solution space with local optima, and more fatally, faces an intractable non-uniqueness issue. It means that diverse metasurface settings can produce nearly identical EM environment (many-to-one mapping). However, deep learning typically optimizes a task with one-to-one mapping, where a single “correct” answer is allowed for each output. When there are more-than-one correct answers for a single input, deep learning will get stuck at how to optimize the weight, making the neural network difficult to converge. Practical randomness and complex EM wave-metasurfaces interaction will make non-uniqueness phenomenon ubiquitous. Standard neural networks produce deterministic predictions and thus remain out of reach for this issue.

There are some approaches proposed to alleviate this challenge. For example, by attaching a pretrained forward network to the end of an inverse design network, tandem neural network can relieve the convergence requirement and optimize the inverse network towards one of the correct directions^63,64^. It has hitherto been used widely in metasurfaces and multi-layer photonic structures. Notice that tandem neural network can mitigate the non-uniqueness issue but does not fundamentally solve it. Deep generative models, e.g., variational auto-encoder (VAE) and generative adversarial network, offer a probabilistic representation to generate a constellation of metasurfaces candidates satisfying the same requirements^65,66^. They first encode the input and output into a latent space, which is then sampled for inverse generation. Another method is mixture density network that models the final output as a multimodal probability distribution, proving the user with many alternatives without sacrificing degenerate solutions^67^.

### Synthetic neural network

**Fig. 4:**
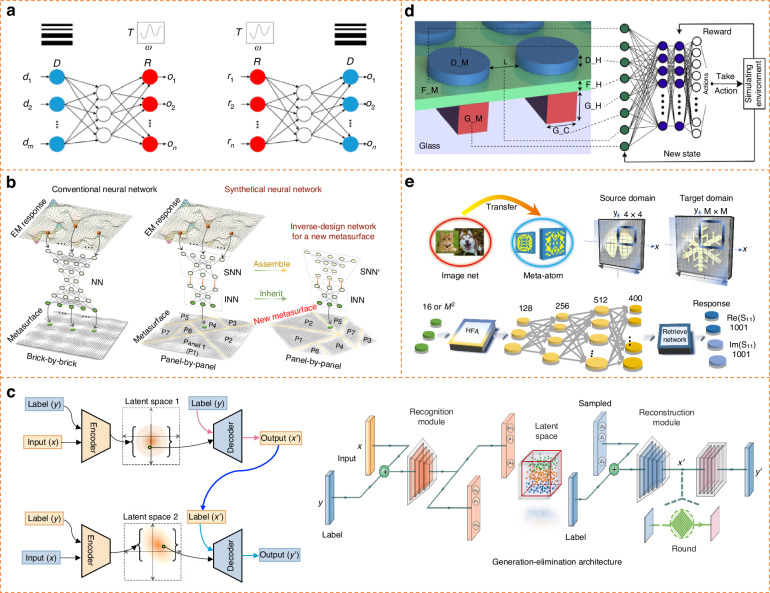
Novel deep learning algorithms to drive intelligent metasurfaces. **a** Tandem neural network by connecting forward and inverse neural network together, aiming to solve non-uniqueness issue in inverse design^63^. **b** Synthetic neural network. The free assembly of metasurfaces in physical space corresponds to the dynamic synthesis of neural networks in algorithm space^68^. **c** Generation-elimination network^71^. Such network firstly generate diverse nominations and then eliminate inferior nominations. **d** Reinforcement learning^74^. **e** Transfer learning to migrate knowledge from old to new task^80,82^. **a** Reprinted with permission from ref. ^63^, Copyright © 2018, American Chemical Society. **b** Reproduced with permission from ref. ^68^, Copyright © 2023, Yuetian Jia et al. **c** Reproduced with permission from ref. ^71^, Copyright © 2023, Jieting Chen et al. **d** Reproduced with permission from Ref. ^74^, Copyright © 2019, Iman Sajedian et al. **e** Reproduced with permission from ref. ^80^, Copyright © 2021, Ruichao Zhu et al, and ref. ^82^, Copyright © 2022, John Wiley and Sons

According to application requirements, free removal and addition of metasurfaces are highly demanded. However, the slight change of whole metasurfaces system will bring in the re-collection of datasets and the re-training of network. It is extremely inefficient because old datasets and networks must be discarded. An ideal practice should endow the neural network with the ability of free assembly and systematic recombination. We hope the slight change of physical space only yields a slight modify of network space. Recently, a knowledge-inherited paradigm, also termed as synthetic neural network and modular neural network, is proposed to allow knowledge inheritance from “parent” to “offspring” metasurfaces^68–70^. “Parent” metasurfaces are defined in advance, linked with a “parent” neural network. Then, for a given “offspring” metasurface, “parent” metasurfaces can be freely assembled to construct it in physical space, corresponding to the dynamic synthesis of “parent” neural networks in algorithm space (Fig. 4b). It is analogous to the building of a container-type house. Such knowledge-inherited paradigm saves a gigantic amount of training data and extends for multi-object, shape-unbound metasurfaces, considerably enhancing the adaptability.

### Semi-known inputs

In 5G wireless communication, an important fact is that the practical input is always semi-known, i.e., information pollution, incomplete and vague input. In this case, how to formulate a suitable input for intelligent metasurfaces plays a key role. For instance, to engineer the beaming towards a user, the perfect input shall be a delta function. However, it is almost impossible to happen in practice. Even if we input the delta function into a pre-trained neural network, it does not mean the output is the optimal. One may artificially repair the delta function with different widths at half maxima and side lobes, as long as the main lobe is fixed. We can easily find that this semi-known problem incorporates infinite possibilities. To solve this, a generation-elimination network is proposed^71,72^, which can orient to the best output candidate. The network consists of two VAE cascaded networks, namely, generation and elimination networks (Fig. 4c). First, the generation network produces many candidates by sampling over its latent space. Then, the elimination network eliminates all suboptimal answers through the merging of two latent spaces; such process is similar to a hierarchical bifurcating tree, a mutual selection between two spaces. Reference. ^73^ has experimentally validated the idea in intelligent metasurfaces that automatically meet the semi-known beam steering.

### Reinforcement learning

Reinforcement learning was born for decades and widely adopted to a wide range of sequential decision-making tasks, exemplified by board games. It enables a computer agent to assimilate the parameter space of an environment with the goal of reward maximum. Distinct from the training of other neural networks, the output reward only needs to be computed at the state when the agent visits, allowing optimization without dense sampling of the entire parameter space^74–76^. High-frequency wireless communications, such as mmWave, suffer from a high pathloss due to Friis’ equation and is vulnerable to obstruction, leading to information outage and intermittent connectivity. Most existing works are based on a simplified channel model, which cannot exactly characterize the channel and reflect practical cases. Current machine learning-based works heavily rely on the environment-dependent channel characteristics. In this case, reinforcement learning can leverage its maximum advantage to achieve robust performance in dynamics, such as the phase shifts design for multiple-input single-output wireless transmission system^77^ and smart beamforming at the base station against an eavesdropper^78^.

### Transfer learning

Transfer learning is a widely-used algorithm in computer science. The basic process is to transfer the pre-trained neural network in the source task to assist the training of target task. The biggest advantage is to relieve the demand for labeled input and accelerate the training process^79,80^. In wireless communication, it would be very useful to implement scenario knowledge transfer and predict channel modeling. For example, transfer learning can save 30% data for inverse metasurface design^81^. However, the performance of transfer learning is instable to some extent. It behaves like a ‘black box’ without unlocking the internal mechanism, which heavily depends on the brute-force attack of features and thus lacks reasonable explanation. Why different scenes can use transfer learning? Why this scene performs better than another scene? These questions are still elusive. To enhance the performance and robustness, heterogeneous transfer learning and attention-based transfer learning are worthy of further explorations and provide physical extrapolation^82^.

## Experimental implementations

Prototypes of intelligent metasurfaces have been experimentally fabricated to showcase the feasibility of editing wireless communication environment. Far beyond the basic functionality of wavefront shaping (signal relay), researchers find that intelligent metasurfaces can be leveraged to implement more complex tasks, such as simplified transmitter based on temporal modulation, and wave-based signal analogous computing. In the following, we follow the framework of signal relay, signal transmitter, and signal processor.

### Signal relay

The popular utilization of intelligent metasurfaces is to counteract localized coverage holes, where the signal strength is relatively low. In both densely populated cities and indoor environments, such as industrial factories and underground metro stations, dead zones widely exist. Therefore, passive/active/intelligent metasurfaces can be decorated to the facades of surrounding objects, such as the walls and ceilings, and even carried by aerial platforms, such as floating balloons and unmanned aerial vehicles (UAVs). Each metasurface behaves like a signal relay to reflect the incident signal, and the global contribution of multiple metasurfaces ultimately strengthen the signal in dead zones. In essence, metasurfaces play a role of EM wave manipulator, which has been widely demonstrated in electromagnetism and optics in past decade, including near-perfect absorption, beam splitting, polarization converter, anomalous reflection, invisibility cloak, and so on^83–88^. Having the basic proof-of-concept validations, the extension to the field of wireless communication is not very challenging.

Initial demonstrations of basic wavefront shaping enabled by metasurfaces are numerous, covering both passive and active manner, spanning from microwaves, optics, to acoustics, and water waves^89–92^. For example, the authors in ref. ^93^. proposed the concept of coding metasurfaces and experimentally demonstrated the far-field engineering using 1-bit PIN diode integrated metasurfaces. The authors in ref. ^94^ implemented a metasurface containing 1600 individually unit cells to demonstrate the dynamic wave manipulations. Each unit cell is integrated with PIN diode that can be switched between ON/OFF states. The authors in ref. ^95^ developed large-scale THz all-dielectric metamaterials with the outer dimension of 900 cm × 900 cm. Based on such metamaterials, the authors implemented a broadband reflector with a bandwidth of 0.15 THz and demonstrated its reflection up to 95%. Many reviews on wavefront shaping have been made in the context of electromagnetism, optics, and wireless communications; the interested reader is directed towards many literature^19^.

**Fig. 5:**
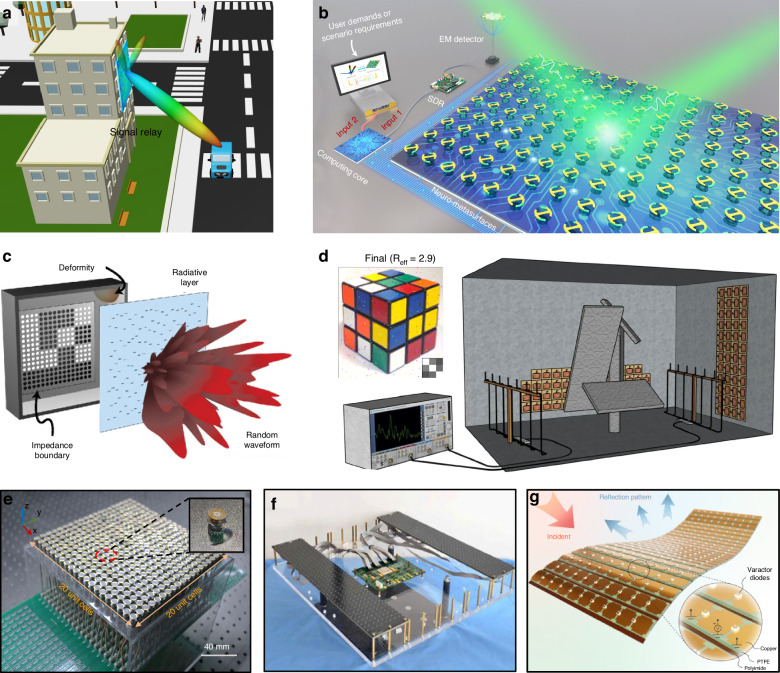
Signal relay enabled by intelligent metasurfaces. **a** Schematic illustration of signal relay. **b** Perception-decision-execution intelligent metasurfaces for wireless channel management^98^. **c** Tunable impedance surface in disordered cavity^99^. **d** Optimal channel diversity in disorder^97^. **e** Mechanical-actuating metasurfaces^98^. **f** Tunable metasurfaces embedded with PIN diodes^94^. **g** Conformal reconfigurable metasurface^100^. **b**, **e** Reproduced with permission from ref. ^98^, Copyright © 2022, American Association for the Advancement of Science. **c** Reproduced with permission from ref. ^99^, Copyright © 2016, American Physical Society. **d** Reproduced with permission from ref. ^97^, Copyright © 2019, Springer Nature. **f** Reproduced with permission from ref. ^94^, Copyright © 2016, Huanhuan Yang et al. **g** Reproduced with permission from ref. ^100^, Copyright © 2023, Erda Wen et al

When truly applying and discussing metasurfaces for wireless communications, the related works starts just a few years ago. Arun et al. built an RFocus prototype with 3200 passive meta-atoms on a six square-meter surface, which moves beamforming functions from the radio endpoints to the environment (Fig. 5g). The states of the meta-atoms are optimized to improve the median signal strength by 9.5×^96^. Hougne et al. deployed 1-bit tunabled metasurface to alter the boundary conditions of the environment. They carried out microwave experiment in an office to achieve completely independent channels and improve the equivalent number of channels of MIMO wireless communication^97^, as shown in Fig. 5d. Fan et al. introduced homeostatic perception-decision-execution neuro-metasurfaces to monolithically manage wireless channel in the dynamic propagation^98^. A mechanical-actuating neuro metasurface is proposed, whose reflection phase is independently controlled by mechanical rotation (Fig. 5e). Mechanical neuro-metasurfaces execute geometric actuation without continuous energy supply (non-volatile); the heat dissipation issue would be alleviated. Even more speculatively, the study of intelligent metasurfaces is promising for many other applications, including target localization and electronic surveillance^99,100^. Overall, there are rapid development in this topic, refs. ^5,6^. provide more comprehensive reviews about signal relay.

### Signal transmitter

Conventional wireless communication systems consist of miscellaneous RF components, including oscillators, mixers, and power amplifiers. There are challenges associated with signal generation: high-cost and heavy hardware, power consumption, and spectrum allotment. Intelligent metasurfaces provide new route for simplified-architecture transmitters and information modulations. The traditional RF components are replaced by tunable metasurface, with the advantages of low cost, easy assembling, and low power consumption. A simple example is the direct transmission of multichannel data after optimization of scattering environment^101^. For example, optimizing the phase distribution of single-layer tunable metasurfaces to allow multiple independent channels in that way of wave focusing points^102^. In ambient environment, experiment demonstrates three-channel backscatter wireless communication with the commodity wireless fidelity (Wi-Fi) signals^103^.

Furthermore, when embedding temporal modulation to spatially-modulated metasurfaces, conventional tunable metasurfaces will be updated to spatiotemporal metasurfaces, capable of manipulating EM signals in both frequency and space domain^104–109^. Note that temporal modulation here indicates the modulation speed is comparable with the incident frequency, while the speed of conventional tunable metasurfaces is too low to be defined. Illuminated under a plane wave, spatiotemporal metasurfaces can generate a lot of harmonic waves by feeding periodical time-varying series. Based on Fourier theory, periodic square-wave series can be synthesized by a series of orthogonal sine functions with different angular frequencies. In other simple way, spatiotemporal metasurfaces can synthesize a number of equivalent reflection states, beyond original discrete states provided by tunable metasurfaces. Now, spatiotemporal metasurfaces have shown the potentials in the binary frequency shift keying transmitter, quadrature amplitude modulation transmitters, and so on^110–118^.

**Fig. 6:**
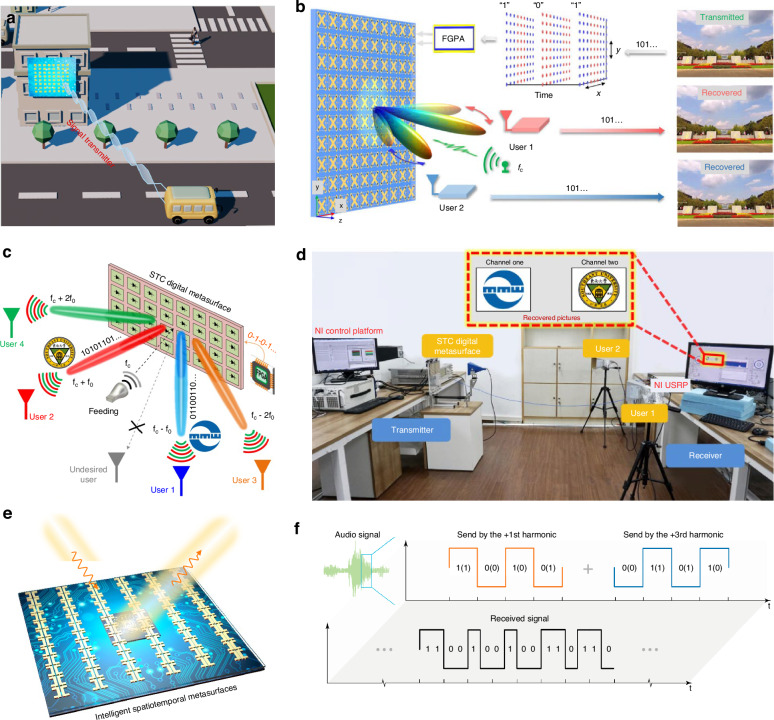
Signal transmitter enabled by intelligent metasurfaces. **a** Schematic illustration of signal transmitter. **b** Broadband wireless communication based on the space-time-varying metasurfaces^105^. **c** Space- and frequency-multiplexing wireless communications^112^. **d** Experimental setup of the dual-channel wireless communications^112^. **e** Intelligent spatiotemporal metasurfaces^113^. **f** Index modulation^113^. **b** Reproduced with permission from ref. ^105^, Copyright © 2023, Qi Hu et al. **c**, **d** Reproduced with permission from ref. ^112^, Copyright © 2021, Springer Nature. **e**, **f** Adapted with permission from ref. ^113^, Copyright © 2023, Xiaoyue Zhu et al

Figure 6b, c showcases a wireless communication with both space- and frequency-division multiplexing enabled by spatiotemporal metasurfaces^112^. Spatiotemporal metasurfaces are realized by feeding different time-varying signals to dynamically modify the working state. Two users at different locations are assigned with different harmonic frequencies. Specific time-varying sequences are firstly optimized for a certain surrounding environment. As a demonstration, two pictures were encoded and then translated into time-varying sequence that is fed into tunable metasurfaces. Thus, the carrier signal emitted from a feeding horn antenna was modulated, and then the reflected signals are individually received by two horn antennas and then recovered to obtain the two pictures.

Another example is to experimentally implement index modulation, a modulation method to relax the increasing demands on escalating tele-traffic^113^. Theoretically, it has been proved that index modulation is capable of reducing bit error rates and enhancing spectral efficiency and anti-interference ability because part information (e.g., subcarriers, antennas, and time slots) is implicitly embodied into the transmitted signal. Spatiotemporal metasurfaces provide a promising platform to realize index modulation. Different harmonic waves can be generated and leveraged to provide additional bits for the index modulation. To intellectualize the index modulation, the authors adopt tandem neural networks to connect the propagation route of harmonic waves with the time-varying sequences. In the experiment, the authors discuss how to generalize the input for vague target and consider two multiuser scenes to demonstrate the index modulation.

### Signal processor

Metasurfaces have recently found to provide a wave-based platform to execute a series of mathematical operations and information processing at the physical layer^119–137^. Thanks to the distinct features of ultra-fast signal computation and low power consumption, wave-based processing holds huge potential in a myriad of real-world scenarios involving high-throughput, on-the-fly data, and scheduled tasks. At the front end of conventional wireless communication system, metasurfaces can be leveraged to implement signal pre-processing, such as encoding, dimensionality reduction, detection, and so on. Metasurfaces are extremely suitable at linear computations, while electronic processors are good at nonlinear operations. The cooperation between metasurfaces and RF components, and hybrid computation between EM waves and existing electronic computational technologies, can maximize respective advantages to foster a more efficient and rapid information processing system. Recent years have seen a proliferation of works, encompassing the exploitation of the linear and nonlinear properties of light to undertake complex computational tasks.

**Fig. 7:**
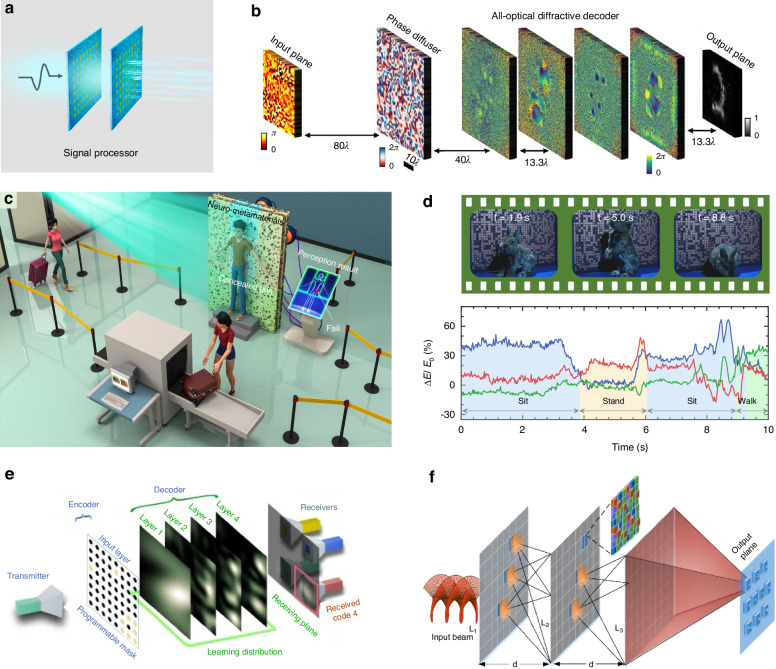
Signal processor enabled by intelligent metasurfaces. **a** Schematic illustration of signal processor. **b** All-optical diffractive decoder^133^. **c** Neuro-metasurfaces for signal processing^135^. **d** Dynamical recognition by neuro-metasurfaces^135^. **e** Multi-user information propagation^131^. **f** Orbital angular momentum detection^136^. **b** Reproduced with permission from ref. ^133^, Copyright © 2023, Yuhang Li et al. **c**, **d** Reproduced with permission from ref. ^135^, Copyright © 2022, Chao Qian et al. **e** Reproduced with permission from ref. ^131^, Copyright © 2022, Springer Nature. **f** Reproduced with permission from ref. ^136^, Copyright © 2019, Elsevier

Here we showcase some examples of signal processor based on metasurfaces, in particular, cascaded metasurfaces. First, metasurface encoder and decoder. Feature dimensionality reduction and recovery are a necessary process for image segmentation and computer vision. Convolutional autoencoder (CAE) has been widely applied to capture local spatial features in images, yielding a more efficient low-dimensional representation for subsequent tasks. Using metasurfaces to mimic the functionality of CAE is a doable way to encode high-dimensional data at the physical layer^132^. Refs. ^133^. demonstrated jointly-optimized electronic encoder and all-optical diffractive decoder for optical information transfer through random unknown diffusers (Fig. 7b). Second, diffractive neural network constructed with cascaded metasurfaces can manipulate vortex beams by carefully designing the phase and amplitude distribution of diffractive screens^134^. In that work, diffractive neural network is theoretically trained to demonstrate hybrid-orbital angular momentum (OAM)-mode generation, identification, and conversion, and three classical optical communication applications (OAM-shift keying, OAM multiplexing and demultiplexing, and OAM-mode switching).

Another important application is the detection and recognition by directly analysing the scattering waves^135^. Figure 7c shows the schematic of neuro-metamaterials for 3D object by harnessing the metamaterial elements to offer subwavelength scattering elements as training parameters. Neuro-metamaterials are optimized using diffractive neural network and fabricated using high-efficiency transmitted metasurfaces. In the experiment, a living rabbit freely plays in front of the neuro-metamaterials without human intervention, and its postures can be detected with light speed (Fig. 7d). The resulting neuro-metamaterials exhibit the “inherent intelligence” to perform complex tasks and brings wave-based computing closer to practical scenes. Figure 7f shows the detection of OAM with transmissive diffractive layers^136^. The simulation results show that the diffractive layers can classify ten types of multiplexed OAM modes under different strength turbulence conditions. Polarization plays a crucial role in electromagnetism and optics. Detecting the polarization state is required in remote sensing, material analysis, and biology. However, conventional methods necessitate the cascading of multiple bulky optical devices and consequential measurements to estimate the Stokes parameters. A simple strategy is recently introduced for direct readout of polarization using dual-channel neuro-metasurfaces^137^. Neuro-metasurfaces independently manipulate two orthogonal linearly-polarized waves that can synthesize arbitrary polarization waves. Through carefully designing the output focusing points, a unique polarization atlas is created to allow one-to-one correspondence between intensity ratio andpolarization state.

## Outlook

Despite the great successes made in the theorical modeling and proof-of-concept experiments (Table 1), this emerging concept still faces multifaceted challenges that impede the further off-the-shelf applications. For example, a majority of the existing researches largely simplify the intelligent metasurfaces as a diagonal matrix with user-defined phase shifts, while ignoring the physical factors of metasurfaces themselves, such as the coupling among meta-atoms^138^, the phase coverage, and so on. In the distinct future, these problems deserve further investigation.

**Table 1:** Summary of intelligent metasurfaces

Functionality	Algorithm	Tunning mechanism	Dimension	Hardware	Frequency	Polarization	Ref.
Self-adaptive invisibility cloak	Machine learning	Varactor diode	24 × 28 cells (1D)	ARM	6.7–9.2 GHz	Single	^20^
Beam steering and focusing	Genetic algorithm	PIN diode	1600 cells (2D)	FPGA	11.1 GHz	Single	^94^
Wireless channel management	Global inverse design	Micromotor	20 × 20 cells (2D)	STM32	13.4 GHz	Single	^98^
Index modulation	Tandem neural network	PIN diode	8 × 8 cells (1D)	FPGA	3.5 GHz	Single	^113^
Three-dimensional Invisibility cloak	Hybrid inverse design	PIN diode	892 cells (2D)	Jetson	4–6 GHz	Full	^24^
Diverse communication channels	Iterative sequential	PIN diode	102 cells (2D)	Arduino	2.47 GHz	Single	^97^
Backscatter wireless communication	Optimization	PIN diode	24 × 32 cells (2D)	FPGA	2.4 GHz	Single	^103^
Contactless in-home monitoring	Machine learning	PIN diode	48 × 48 cells (1D)	FPGA	3.75 GHz	Single	^118^
Multiplexed wireless communication	Binary particle swarm	PIN diode	16 × 8 cells (1D)	FPGA	9.5 GHz	Single	^112^
Broadband wireless communication	Optimization	PIN diode	30 × 30 cells (1D)	FPGA	3.7–5.1 GHz	Full	^105^

## Design of multi-functional metasurfaces

No matter how elegant the theoretical models may be, the practical performance is fundamentally determined by the physical performance of basic meta-atoms. So far, most of intelligent metasurfaces are demonstrated in reflective manner and the available tunable strategies are few. Developing abundant metasurfaces, including transmitted metasurfaces, are important to enable cascaded system and implement on-site radio signal processing^139–141^. In microwave, the commonly used tunable metasurfaces are embedded with active components, such as p-i-n diodes, varactor diodes, and varying resistance. It incurs some issues, for example, the reflection states are very few and discrete for p-i-n diode integrated metasurfaces, and the DC voltage should be continuously supplied. Mechanical actuation based tunable metasurfaces are a possible solution worthying exploration^98^ due to the non-volatile and Strong anti-interference ability. Narrow bandwidth and limited modulation speed (tens of MHz) are also bottlenecks for current tunable metasurfaces^142–144^. Thus, different meta-atoms need to be configured together when dealing with different working band requirements. Moreover, extending the working frequency to mmWave and terahertz is a future development trend, putting forward a higher requirement for new reconfigurable meta-atoms. From this point of functionality, the use of the intelligent metasurfaces as an array of smart reflector will have vast applications in sensing, e.g., indoor positioning and human pose recognition. Therefore, intelligent metasurfaces-assisted wireless systems will not only enhance communication quality but also bestow the opportunities of close human-network interactions.

## Gain metasurfaces

The initial motivation of using intelligent metasurfaces comes from the ability of manipulating EM waves to edit the wireless communication environment. In a real-world environment, the signal experiences complex reflections, transmissions, and diffractions with attenuated and time-delayed copies of the original signals along different paths. Even when applying intelligent metasurfaces, multiple reflections among them also suffer from propagation loss. Moreover, metasurfaces themselves are inherently a lossy system due to the materials and resistance loss, especially for reconfigurable metasurfaces. Gain metasurfaces are very necessary in this case by amplifying EM waves and enhancing signals. In a traditional and engineering way, there are examples incorporating lumped amplifiers in microwave metasurfaces, which heavily relies on complex structures and additional receiving-transmitting circuits^145^. They are too sophisticated to be widely adopted. Recently, a promising method is proposed by employing tunnel diode^146,147^. The property of negative resistance makes us able to regulate the reflection/transmission amplitude larger than unity, consequently leading to the tunability of the Poynting vector. It has been validated for breaking the fundamental scattering limit.

## Distributed metasurfaces deployment and optimization

The ultimate stage of smart and controllable radio environment requires the dense deployment of passive/tunable metasurfaces by exploiting their multi-hop reflections. This thus calls for new researches to devise innovative solutions for distributed metasurfaces beamforming, channel estimation, detection, and deployment^148–154^. How to determine the number, deploy the location, and design the phase distribution of metasurfaces becomes extremely important. Purely using subjective intuition and past experience may be not enough because it is innately flawed in a trial-and-error manner. Brute-force search calls for a gargantuan number of time-consuming EM numerical simulations and inevitably encounters many failed attempts, causing a great waste of computing and human resources. Practical scenes and future communications are not only diverse but also easily changing, for example, UAVs, as a flexible communication platform, have been found to assist terrestrial wireless communications. As such, developing a generalized intelligent algorithm to handle extremely high degrees of freedom is highly demanded. Compared with image recognition and decision-making, a distinct point for metasurfaces is that they are moderately governed by certain physical laws and semi-analytical solutions. In this logic, deep learning can be combined with physical laws to simplify the modeling of deep learning and enhance physical explainability^155,156^.

The age of intelligent metasurfaces is just dawning. This Review covers a broad set of research topics regarding intelligent metasurfaces and their applications in wireless networks, from its channel modeling, intelligent algorithms, to experimental implementations. Three main wireless communication applications by intelligent metasurfaces have been summarized, i.e., signal relay, transmitter, and processer. We have identified basic problem models and solution methods in deep learning and hardware implementations, to automatically cater for ever-changing environment. In the end, future trend is discussed to involve physical features of metasurfaces, as well as the engineering problems in wireless communication.
